# Supplementary material for: Multi-omic profiling of the leukemic microenvironment shows bone marrow interstitial fluid is distinct from peripheral blood plasma
Source: Exp Hematol Oncol. 2022 Sep 15;11:56. doi: 10.1186/s40164-022-00310-0 (PMC9476264; doi:10.1186/s40164-022-00310-0)
Supplement: Supplementary file 1 — Additional file 1: Complete (non-cropped) and annotated membrane blot. [file 40164_2022_310_MOESM1_ESM.pdf]

Additional File 1

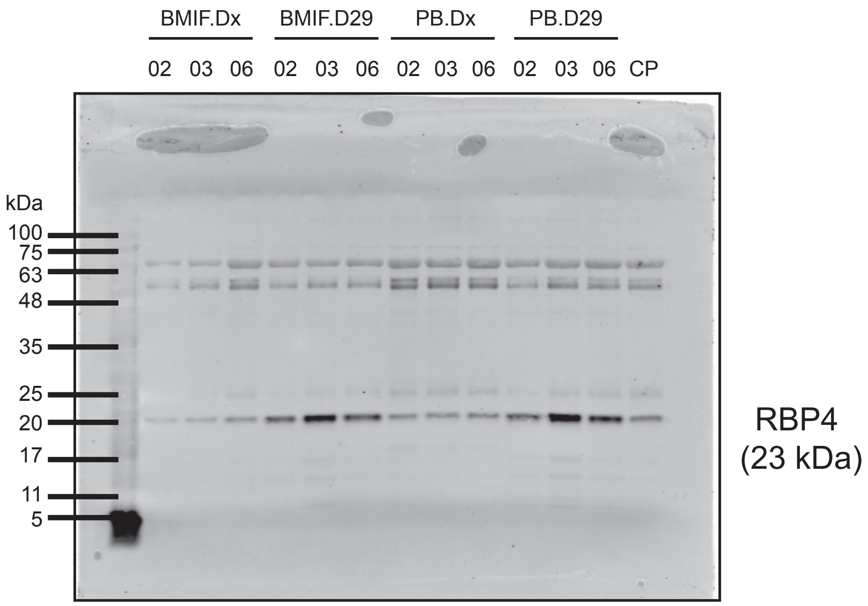

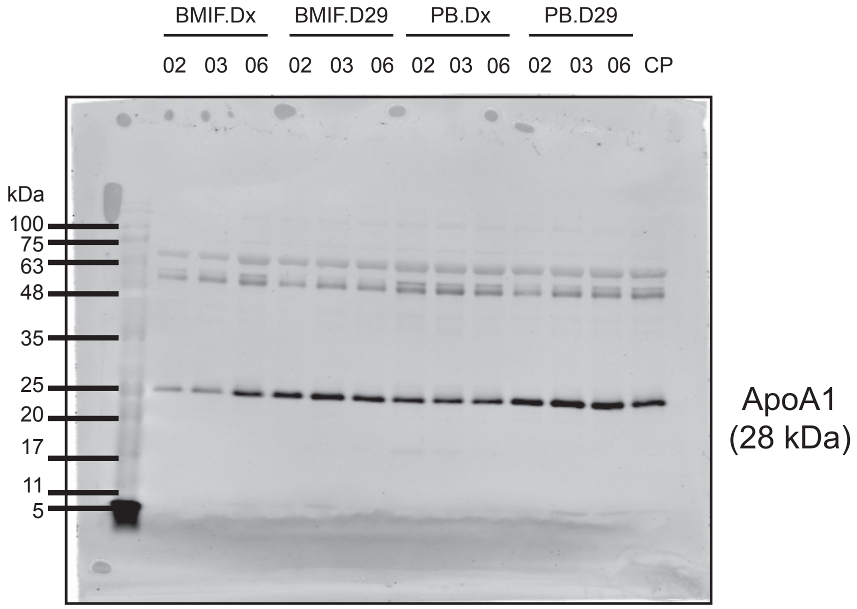

Western blot validation of lipid binding proteins. RBP4 (A) and Apolipoprotein A1 (B) protein levels were confirmed via western blot in patients B-ALL #02, B-ALL #03, and B-ALL #06 across all experimental groups. In addition, a commercially-available human plasma (CP) was also loaded as a healthy control.
