# Supplementary material for: Multi-omic profiling of the leukemic microenvironment shows bone marrow interstitial fluid is distinct from peripheral blood plasma
Source: Exp Hematol Oncol. 2022 Sep 15;11:56. doi: 10.1186/s40164-022-00310-0 (PMC9476264; doi:10.1186/s40164-022-00310-0)
Supplement: Supplementary file 8 — Additional file 8: Table S1. Summary of patient information and clinical data. [file 40164_2022_310_MOESM8_ESM.docx]

Supplementary Table S1. Summary of B-ALL patient information and clinical data for BMIF and PBP studies

| **Patient ID** | **Age (at collection)** | **Sex** | **Risk Group** | **Cytogenetics** | **Treatment** |
| --- | --- | --- | --- | --- | --- |
| B-ALL #01 | 3 years, 2 months | Male | Standard | ETV6/RUNX1 fusion; loss of the native ETV6 and a focal deletion encompassing the PAX5 gene. | Cytarabine, Vincristine, Dexamethasone, Pegasparaginase, Methotrexate |
| B-ALL #02 | 4 years, 2 months | Male | Standard | Translocation (12;21) with fusion ETV6/ RUNX1; complex karyotype; clonal evolution. | Methotrexate, Dexamethasone,Vincristine, Pegasparaginase |
| B-ALL #03 | 9 years, 9 months | Male | Standard | Fusion ETV6/ RUNX1 | Cytarabine, Vincristine, Dexamethasone, Pegasparaginase, Methotrexate |
| B-ALL #04 | 3 years, 6 months | Male | Standard | Hyperdiploid; balanced reciprocal translocation between chromosomes Xq28 and 6p21; trisomies of chromosomes 4 and 10 | Cytarabine, Dexamethasone, Pegasparaginase, Vincristine, Methotrexate |
| B-ALL #05 | 7 years, 8 months | Male | Standard | Complex chromosomal karyotype with evidence of an ETV6-RUNX1 fusion; the presence of ETV6 deletions from the chromosome 12 homologue not involved in the translocation are common secondary changes that accompany the t(12;21); IKZF1 deletions have also been reported in ETV6-RUNX1 fusion positive B-cell ALL. | Cytarabine, Vincristine, Dexamethasone, Pegasparaginase, Methotrexate |
| B-ALL #06 | 15 years, 10 months | Born Female | High | Hypotetraploid; TEL/AML fusion positive clone, with reduced copy number of CDKN2A and fusion of CRLF2-P2RY8 | Cytarabine, Vincristine, Daunorubicin, Pegasparaginase , Prednisone, Methotrexate |
| B-ALL #07 | 4 years, 8 months | Male | Standard | High hyperdiploid clone with segmental loss of CDKN2A | Cytarabine, Vincristine, Dexamethasone, Pegasparaginase, Methotrexate |
| B-ALL #08 | 7 years, 11 months | Female | Standard | Hyperdiploid karyotype; clonal changes | Cytarabine, Methotrexate, Vincristine, Dexamethasone, Pegaspargase |
