# Supplementary material for: Multi-omic profiling of the leukemic microenvironment shows bone marrow interstitial fluid is distinct from peripheral blood plasma
Source: Exp Hematol Oncol. 2022 Sep 15;11:56. doi: 10.1186/s40164-022-00310-0 (PMC9476264; doi:10.1186/s40164-022-00310-0)
Supplement: Supplementary file 9 — Additional file 9: Figure S1. Bone marrow and peripheral blood at diagnosis and after induction therapy. Figure S2. SP3 and HUNTER workflows provide complementary analysis of microenvironment. Figure S3. Lipidomic and metabolic identifications were robust. Figure S4. Differential analysis of lipidomics data. Figure S5. Differential analysis of proteomics data. Figure S6. Western blot validation of lipid binding proteins. Figure S7. Immunosuppressive microenvironment in the after induction therapy. [file 40164_2022_310_MOESM9_ESM.pdf]

Additional File 9: Supplementary figures

Figure S1 | Bone marrow and peripheral blood at diagnosis and after induction therapy.

Figure S2 | SP3 and HUNTER workflows provide complementary analysis of microenvironment.

Figure S3 | Lipidomic and metabolic identifications were robust.

Figure S4 | Differential analysis of lipidomics data.

Figure S5 | Differential analysis of proteomics data.

Figure S6 | Western blot validation of lipid binding proteins.

Figure S7 | Immunosuppressive microenvironment in the after induction therapy.

Supplementary Figure 1

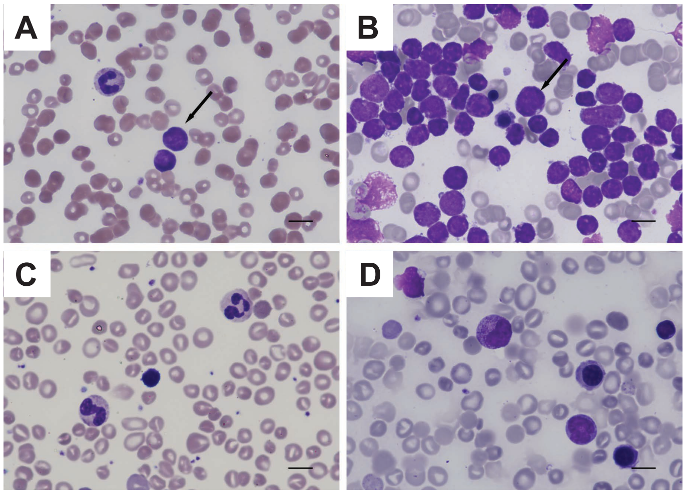

Figure S1. Bone marrow and peripheral blood at diagnosis and after induction therapy. Representative images at diagnosis show blasts (arrows) circulating in the peripheral blood smear **(A)** accompanied with anemia, neutropenia, and thrombocytopenia, while the vast majority of cells in the bone marrow aspirate **(B)** are blasts in a background of reduced trilineage hematopoiesis. Day 29 peripheral blood smear **(C)** shows anisopoikilocytosis with stomatocytes and polychromasia. Day 29 bone marrow aspirate **(D)** shows no definitive blast morphology in a background of increased erythropoiesis and orderly granulopoiesis. All photomicrographs taken at 500x original magnification (oil), scale bar represents 10 microns.

Supplementary Figure 2

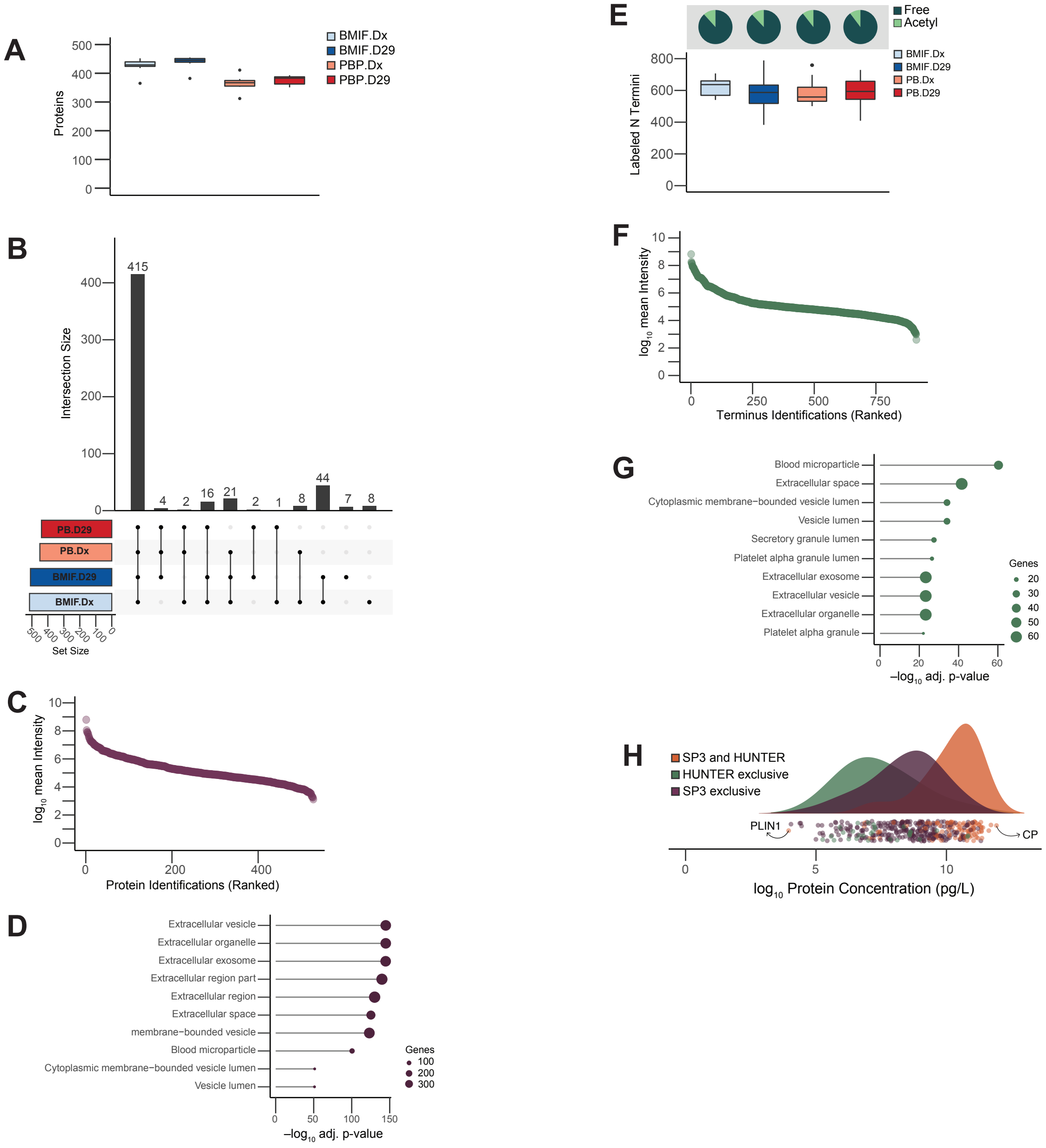

Figure S2 | SP3 and HUNTER workflows provide complementary analysis of microenvironment. (A) Proteomic identifications per experimental group. **(B)** Upset plot for proteomic identifications. Each bar represents the number shared proteins between the indicated experimental groups **(C)** Mean intensity of proteomic identifications. **(D)** Enriched subcellular localizations in COMPARTMENTS analysis of proteomic identifications. **(E)** Terminomic identifications per experimental group. Pie charts specify the fraction of acetylated and unmodified (free) N termini **(F)** Mean intensity of terminomics identifications. **(G)** Enriched subcellular localizations in COMPARTMENTS analysis of proteins in the terminomic identifications. **(H)** Comparison of the expected plasma concentration of SP3- and HUNTER-detected proteins.

Supplementary Figure 3

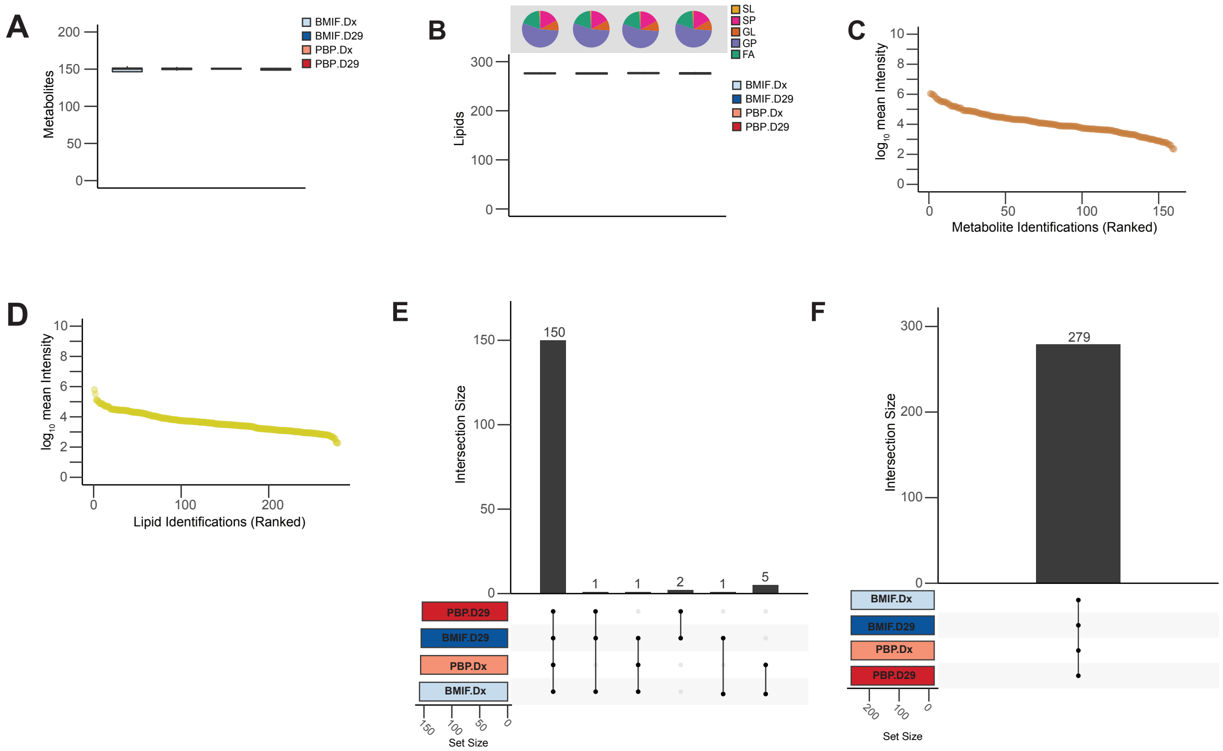

Figure S3 | Lipidomic and metabolic identifications were robust. (A) Metabolomic identifications per experimental group. **(B)** Lipidomic identifications per experimental group. Pie graphs located at the top represent the proportion of different lipid classes per experimental group (SL: sterol lipids; SP: sphingolipids; GL: glycerolipids; GP: glycerophospholipids; FA: fatty acyls). **(C)** Mean intensity of metabolomic identifications. **(D)** Mean intensity of lipidomic identifications. **(E)** Upset plot for metabolomic identifications. Each bar represents the number of shared metabolites between the indicated experimental groups. **(F)** Upset plot for lipidomic identifications. Each bar represents the number of shared lipids between the indicated experimental groups.

Supplementary Figure 4

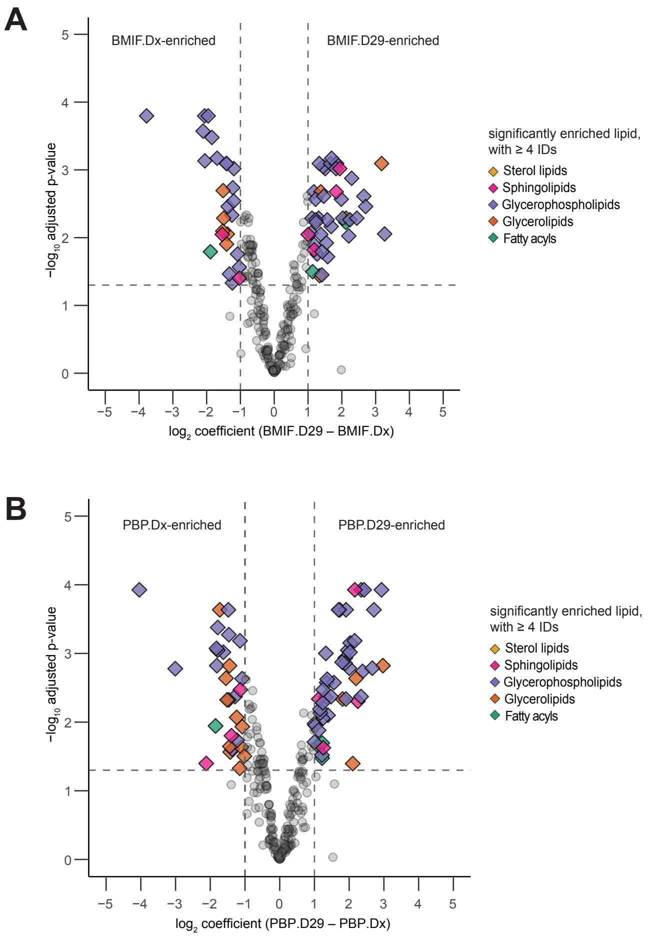

Figure S4 | Differential analysis of lipidomics data. (A) Temporal changes within BMIF (BMIF.Dx vs BMIF.D29). **(B)** Temporal changes within PBP (PBP.Dx vs PBP.D29). Significantly altered lipids are annotated based on lipid class.

Supplementary Figure 5

A

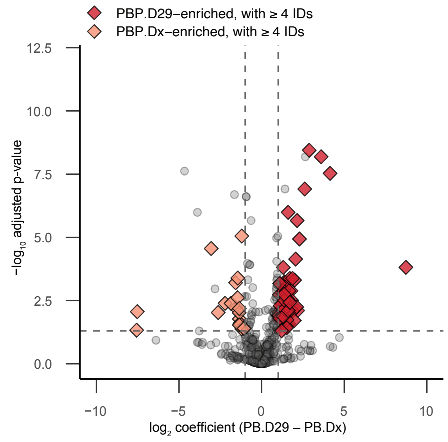

Figure S5 | Differential analysis of proteomics data. (A) Temporal changes within in PBP (PBP.Dx vs PBP.D29).

Supplementary Figure 6

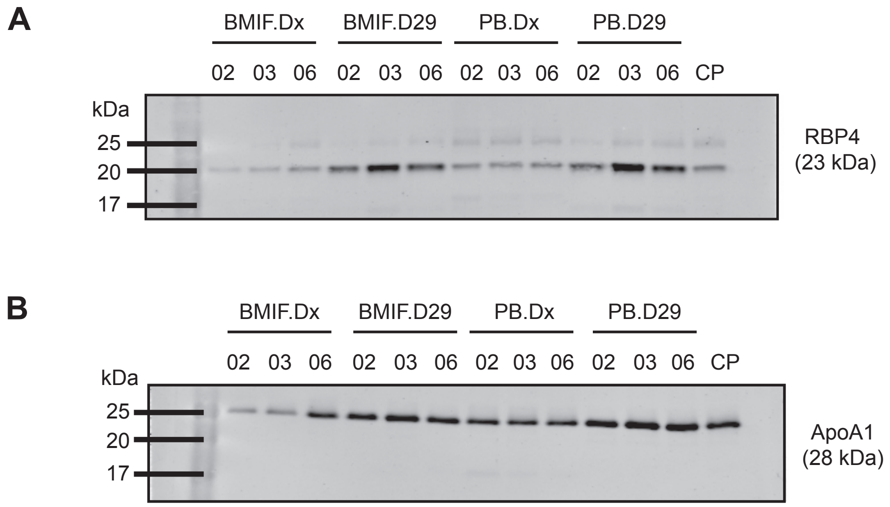

Figure S6 | Western blot validation of lipid binding proteins. RBP4 **(A)** and Apolipoprotein A1 (ApoA1) **(B)** protein levels were confirmed via western blot in patients B-ALL #02, B-ALL #03, and B-ALL #06 across all experimental groups. In addition, a commercially-available human plasma (CP) was also loaded as a healthy control.

Supplementary Figure 7

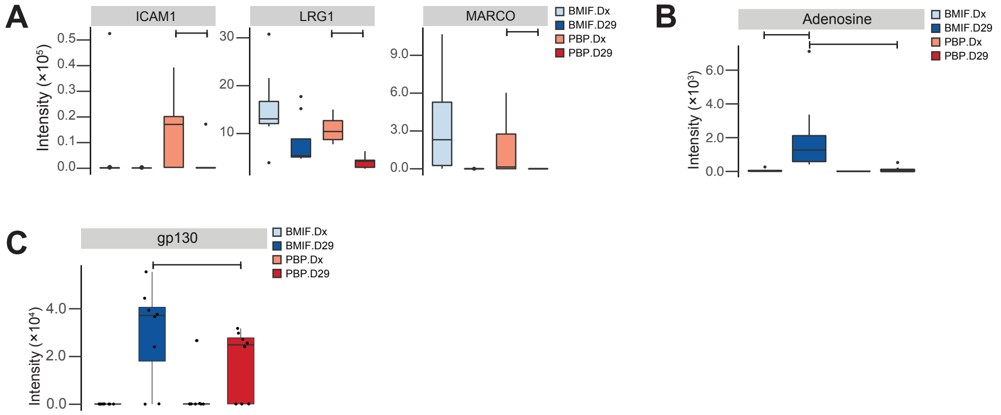

Figure S7 | Immunosuppressive microenvironment in the after induction therapy. (A) Immune proteins with significant declines in intensity after induction therapy based on PBP differential analysis. **(B)** Intensity of adenosine across experimental groups. **(C)** Intensity of gp130/IL6ST across experimental groups. Bars indicate significant difference (limma, adjusted p-value ≤ 0.05) between experimental groups.
